# Estimating burden of influenza‐associated influenza‐like illness and severe acute respiratory infection at public healthcare facilities in Romania during the 2011/12‐2015/16 influenza seasons

**DOI:** 10.1111/irv.12525

**Published:** 2017-12-15

**Authors:** Giedre Gefenaite, Adriana Pistol, Rodica Popescu, Odette Popovici, Daniel Ciurea, Christiaan Dolk, Mark Jit, Diane Gross

**Affiliations:** ^1^ Infectious Hazards Management Division of Health Emergencies and Communicable Diseases WHO Regional Office for Europe Copenhagen Denmark; ^2^ Department of Health Sciences Faculty of Medicine Lund University Lund Sweden; ^3^ National Center for Communicable Diseases Surveillance and Control National Institute of Public Health Bucharest Romania; ^4^ Center for Health Policies and Services Bucharest Romania; ^5^ PharmacoTherapy, ‐ Epidemiology & ‐Economics Groningen Research Institute of Pharmacy University of Groningen Groningen The Netherlands; ^6^ Department of Infectious Disease Epidemiology Faculty of Epidemiology and Population Health London School of Hygiene and Tropical Medicine London UK

**Keywords:** burden, influenza‐like illness, severe acute respiratory infection

## Abstract

**Background:**

Influenza is responsible for substantial morbidity and mortality, but there is limited information on reliable disease burden estimates, especially from middle‐income countries in the WHO European Region.

**Objectives:**

To estimate the incidence of medically attended influenza‐associated influenza‐like illness (ILI) and hospitalizations due to severe acute respiratory infection (SARI) presenting to public healthcare facilities in Romania.

**Patients/Methods:**

Sentinel influenza surveillance data for ILI and SARI from 2011/12‐2015/16, including virological data, were used to estimate influenza‐associated ILI and SARI incidence/100 000 and their 95% confidence intervals (95% CI).

**Results:**

The overall annual incidence of ILI and influenza‐associated ILI per 100 000 persons in Romania varied between 68 (95% CI: 61‐76) and 318 (95% CI: 298‐338) and between 23 (95% CI: 19‐29) and 189 (95% CI: 149‐240), respectively. The highest ILI and influenza incidence was among children aged 0‐4 years. We estimated that SARI incidence per 100 000 persons was 6 (95% CI: 5‐7) to 9 (95% CI: 8‐10), of which 2 (95% CI: 1‐2) to 3 (95% CI: 2‐4) were due to influenza. Up to 0.3% of the Romanian population were annually reported with ILI, and 0.01% was hospitalized with SARI, of which as much as one‐third could be explained by influenza.

**Conclusions:**

This evaluation was the first study estimating influenza burden in Romania. We found that during each influenza season, a substantial number of persons in Romania suffer from influenza‐related ILI or are hospitalized due to influenza‐associated SARI.

## 1 INTRODUCTION

Influenza is a respiratory disease responsible for substantial increase in morbidity, mortality and costs during seasonal epidemics and pandemics.^1^, ^2^, ^3^, ^4^, ^5^, ^6^ The World Health Organization (WHO) estimates that annually seasonal influenza epidemics result in an estimated 3‐5 million cases of severe disease and 250 000‐500 00 deaths.^7^ However, the burden of seasonal influenza can vary between influenza seasons and countries. Therefore, reliable disease burden estimates are needed, especially from low‐ and middle‐income countries to provide a better understanding of the impact of influenza. Knowledge about influenza burden is crucial to further develop and strengthen influenza prevention strategies, as well as to assess utility. Thus, national estimates would enable governments, non‐governmental agencies, and others to make informed evidence‐based decisions when allocating limited resources dedicated to public health and planning intervention strategies to limit the spread of influenza.

While some estimates of the national burden of influenza are available for countries of the WHO European Region, the majority of primary study estimates come from Western Europe and no estimates are available for over half of the member states.^8^, ^9^

Except for a study on the economic influenza burden among the elderly in 6 middle‐income countries of Central and Eastern Europe in 2010/11,^9^ influenza burden in the Romanian population has never been estimated.

To gain insight into medically attended influenza burden on a national level, we performed an assessment of influenza disease burden due to influenza‐like illness (ILI) attendances and severe acute respiratory infection (SARI) hospitalizations associated with influenza in Romania at public facilities for the 2011/12‐2015/16 influenza seasons.

## 2 METHODS

**Figure 1:**
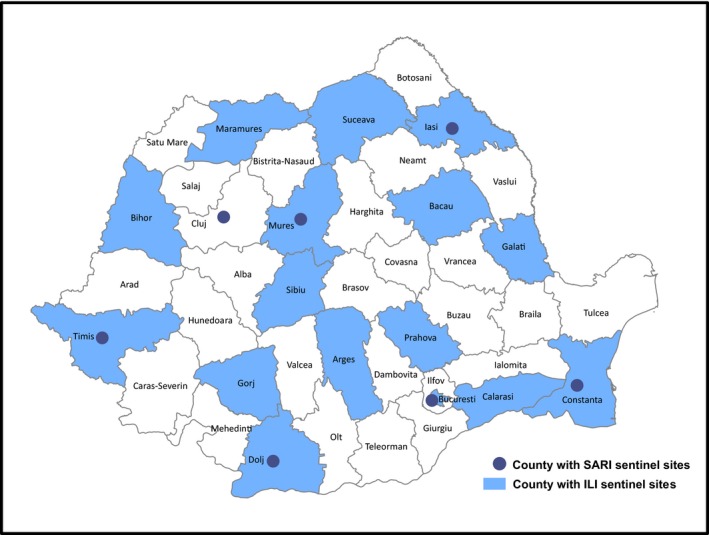
Influenza‐like illness (ILI) and severe acute respiratory infection (SARI) sentinel surveillance counties in Romania in 2015/16 influenza season

This study followed the methods outlined in the WHO Manual, A Manual for Estimating Disease Burden Associated with Seasonal Influenza in a Population, using 5 years of Romania national surveillance data.^10^ The WHO European Region lies in the Northern Hemisphere, and the influenza season is considered to be from calendar week 40 to week 20 of the following calendar year.^11^ Romania performs influenza sentinel surveillance for ILI from week 40 to week 20 and for SARI from week 46 to week 20. Anonymous, routinely collected, retrospective national sentinel surveillance data on ILI and hospitalized SARI cases, including influenza‐associated deaths among SARI cases, were analysed for the 2011/12‐2015/16 influenza seasons. This included virological information obtained from testing of nasopharyngeal swabs for influenza virus using reverse transcription‐polymerase chain reaction (RT‐PCR). For the overview of the geographical distribution of the counties contributing to the ILI and SARI influenza sentinel surveillance, see Figure 1.

### 2.1 Influenza‐like illness sentinel surveillance and catchment population

From 2011/12‐2013/14 influenza seasons, sentinel ILI surveillance was conducted in all 41 counties and the capital city Bucharest. This included at least 270 general practitioners (GPs) from urban and rural areas with both adult and paediatric patients. This represented 1% of the GPs and covered approximately 2% of the population in Romania. These GP practices were selected by the counties' public health authorities and agreed to participate. Since 2014, sentinel ILI surveillance is performed in 15 counties and Bucharest and included at least 192 GPs. Cases were identified from patients who sought care at the clinic and those receiving home visits. In line with the WHO definition, ILI was defined as an acute respiratory infection with measured fever of ≥38°C and cough, with onset within the last 10 days.^12^ Samples for virological testing were collected 1 day per week (first two cases at each participating GP practice every Tuesday). Samples were only collected from ILI cases belonging to groups targeted for vaccination, including pregnant women, persons with underlying chronic conditions, aged >=65 years, HCWs, residents of long‐term care facilities. In Romania, the majority of the population is covered by a universal health insurance and registered with a GP practice. As these patients must seek care from the GPs with which they are registered, the catchment population was estimated by summing the patients registered at each participating GP practice.

### 2.2 Severe acute respiratory infection sentinel surveillance and catchment population

Since 2009, hospital‐based sentinel surveillance for SARI in Romania has been conducted in 20 university hospitals in 7 counties from all 4 geographical regions (Figure 1).

A SARI case was defined as an acute respiratory infection with an onset in the previous 7 days requiring overnight hospitalization, history of fever or measured fever ≥38°C, cough and shortness of breath or difficulty breathing.^11^, ^12^ Starting with the 2013/14 season, the case definition was expanded to include an onset within the previous 10 days. Information on pregnancy status, underlying medical conditions and the outcome of the illness was also recorded for each SARI case. A subset of SARI cases was tested for influenza virus using RT‐PCR. The sampling algorithm for SARI cases had a two‐step approach with greater sampling early in the influenza season and decreased once influenza circulation had been identified. Starting in week 46, the first three identified SARI cases in each sentinel hospital were sampled per week. This sampling strategy continued until ≥10% of all the sentinel surveillance system samples (ILI and SARI) tested positive for the same influenza subtype/variant. Once the positivity rate by subtype/variant reached 10%, only the first identified SARI case in each sentinel hospital each week was sampled for the remainder of the influenza season.

Because the catchment population for each hospital was unknown, an estimate was made by dividing the total number of all‐cause hospitalizations for each sentinel hospital by the total discharges (including deaths) from all the public hospitals in Romania to determine the proportion of all‐cause discharges for each sentinel hospital. This proportion was used as a proxy for the proportion of the population that sought care at each hospital and it was then multiplied by the Romanian population for each year to determine the catchment population for each sentinel site. The catchment population for all sites was summed to produce the estimate of the catchment population of all sentinel hospitals.

### 2.3 Statistical analysis

To estimate the annual incidence of ILI and SARI by type and subtype, we summed the weekly incidence in the sentinel sites of each influenza season and calculated their 95% confidence intervals by 1) calculating the error factor (EF), which was the exponentiation of 1.96 divided by the square root of number of (influenza‐associated) ILI or SARI cases, and 2) calculating the range of the 95% CI by dividing (higher CI) or multiplying (lower CI) the incidence by the EF, as described in WHO Manual.^10^

We stratified the ILI and influenza‐associated ILI incidence by age into 0‐4 years, 5‐14 years, 15‐64 years and 65 years and older groups. Due to limited availability of the hospital discharge data by different age groups, we were only able to stratify SARI and laboratory‐confirmed influenza‐associated SARI cases into paediatric (<16 years) and adult (≥16 years) age groups. All incidences were extrapolated to the Romanian population.

We calculated case fatality proportion (CFP, %) among laboratory‐confirmed influenza‐associated SARI cases by dividing the number of laboratory‐confirmed influenza‐associated deaths among the SARI patients by the total laboratory‐confirmed influenza‐associated SARI cases and multiplying it by 100%. We estimated its 95% confidence interval by the Clopper‐Pearson method.^13^

The Romanian population size by age groups was extracted from the National Institute of Statistics in Romania as of January 1 for the years 2011‐2016 (http://www.insse.ro/cms/en). Approximately, 5% of the Romanian population was 0‐4 years old, 11% was 5‐14 years old, 68% was 15‐64 years old, and 16% was 65 years and older.

### 2.4 Ethical issues

The project was reviewed by the Ministry of Health of Romania and judged to be a public health evaluation. Only anonymized records were used for the analysis.

## 3 RESULTS

During the study period from the 2011/2012‐2015/2016, Romania experienced three seasonal influenza epidemics predominately due to influenza A virus and two seasons due to both influenza A and B (Table 1). One season was dominated by influenza A(H3) and two seasons by influenza A(H1N1)pdm09.

**Table 1:** Influenza by (sub)type, ILI and catchment population of the sentinel surveillance system by age in Romania in 2011/12‐2015/16 influenza seasons

Season	Influenzan					ILI casesn (% of the total ILI cases)					Catchment populationN				
	A(H1N1)pdm09	A(H3)	B	Total specimens	Total positives n (%)	0‐4 y	5‐14 y	15‐64 y	≥65 y	Total	0‐4 y	5‐14 y	15‐64 y	≥65 y	Total
2011/12	0	182	1	668	183 (27.4)	184 (23.1)	164 (20.6)	380 (47.6)	70 (8.8)	798	45 620	93 597	432 558	104 487	676 261
2012/13	45	3	78	454	126 (27.8)	134 (20.2)	144 (21.7)	352 (52.9)	35 (5.3)	665	43 789	88 172	431 824	102 810	666 594
2013/14	42	79	3	379	127 (33.5)	94 (20.5)	72 (15.7)	261 (57)	31 (6.8)	458	41 493	85 683	439 629	102 849	669 653
2014/15	56	106	85	445	247 (55.5)	176 (13.4)	399 (30.4)	649 (49.5)	87 (6.6)	1311	23 456	54 348	270 321	62 107	410 231
2015/16	122	18	16	441	156 (35.4)	175 (20.7)	245 (29)	384 (45.5)	40 (4.7)	844	23 002	55 336	271 221	62 390	411 948

ILI, influenza‐like illness.

The incidence of both ILI and influenza‐associated ILI was highest in children, especially children under 5 years of age (Table 2), where approximately 20% of ILI cases occurred (Table 1). The overall annual incidence of medically attended ILI at public GP practices per 100 000 population varied between seasons and was estimated to range from 68 (95% CI: 61‐76) to 318 (95% CI: 298‐338) cases, with the highest and lowest incidence recorded in 2013/14 and 2014/15, respectively (Table 2). When the results were extrapolated to the population, most of the ILI and influenza cases appeared in the 15‐ to 64‐year‐old group, which comprised approximately 68% of the Romanian population (Appendix 1). The lowest burden of ILI and influenza‐associated ILI was estimated in the cases 65 years old and older (Table 2). Influenza A(H1N1)pdm09 was responsible for the highest incidence of influenza‐associated ILI in 2015/16, influenza A(H3) in 2011/12 and 2013/14 and influenza type B in 2012/13 and 2013/14 influenza seasons (Table 2).

**Table 2:** Incidence of influenza by subtype, influenza‐associated ILI and ILI by age group per 100 000 population from sentinel surveillance in Romania in 2011/12‐2015/16 influenza seasons

	Influenza‐associated ILI incidence per 100.000 (95% CI) by (sub)type				Influenza‐associated ILI incidence per 100.000 (95% CI) by age group				ILI incidence per 100.000 (95% CI) by age group				
	A(H1N1)pdm09	A(H3)	B	Total	0‐4 y	5‐14 y	15‐64 y	≥65 y	0‐4 y	5‐14 y	15‐64 y	≥65 y	Total
2011/12	‐	32 (27‐38)	0 (0‐34)	32 (27‐38)	108 (90‐128)	49 (41‐59)	24 (20‐29)	14 (12‐17)	403 (337‐480)	175 (145‐211)	88 (78‐99)	67 (50‐91)	118 (108‐128)
2012/13	12 (8‐18)	1 (0‐8)	23 (18‐31)	36 (4‐331)	82 (66‐102)	68 (7‐621)	31 (3‐286)	10 (1‐92)	304 (247‐375)	162 (132‐198)	81 (72‐92)	34 (22‐53)	99 (91‐109)
2013/14	8 (5‐11)	15 (11‐20)	0 (0‐4)	23 (19‐29)	70 (56‐87)	29 (23‐36)	21 (17‐26)	10 (8‐12)	225 (175‐291)	84 (62‐112)	59 (51‐68)	30 (19‐49)	68 (61‐76)
2014/15	41 (29‐58)	70 (55‐89)	78 (60‐102)	189 (149‐240)	395 (340‐460)	439 (346‐557)	146 (115‐185)	81 (63‐102)	748 (624‐896)	730 (649‐821)	238 (218‐261)	139 (107‐182)	318 (298‐338)
2015/16	53 (43‐66)	5 (3‐11)	12 (6‐24)	71 (58‐86)	207 (170‐251)	139 (115‐169)	56 (46‐68)	22 (18‐27)	759 (633‐911)	443 (380‐515)	142 (126‐160)	64 (42‐96)	205 (189‐221)

ILI: influenza‐like illness.

95% CI: 95% confidence interval.

On average, approximately 27%‐56% of ILI cases in Romania could be explained by influenza (Table 1), and positivity among the SARI cases also varied over influenza seasons 24%‐43% (Table 3). Influenza‐associated SARI incidence was higher among the <16‐year‐old cases, except for 2014/2015 and 2015/16 influenza seasons, when the incidence appeared to be higher in the ≥16‐year‐old cases. Influenza A(H1N1)pdm09 was responsible for the highest incidence in 2012/13 and 2015/16, influenza A(H3) in 2011/12 and 2013/14 and influenza B in 2014/15 season (Table 4). We estimated that annually approximately 1118 (95% CI: 956‐1308) to 2059 (95% CI: 1839‐2304) persons are hospitalized at public hospitals due to SARI, of which 317 (95% CI: 232‐434) to 625 (95% CI 505‐774) are due to influenza (Appendix 2). The CFP for influenza‐positive SARI cases varied from 1% (95% CI: 0‐8) to 40% (95% CI: 29‐51) in 2011/12 and 2015/16, respectively (Table 5).

**Table 3:** Influenza‐positive deaths, influenza‐positive SARI cases, sampled SARI cases and total numbers of SARI cases, SARI catchment population of the sentinel surveillance in Romania by paediatric and adult age groups in 2011/12‐2015/16 influenza seasons

	Influenza‐positive deaths			Influenza‐positive SARI cases			Sampled SARI cases			SARI cases			SARI catchment population		
	<16 ys	≥16 y	Total	<16 y	≥16 y	Total	<16 y	≥16 y	Total	<16 y	≥16 y	Total	<16 y	≥16 y	Total
2011/12	1	0	1	31	38	69	175	108	283	235	111	346	944 865	2 924 105	3 951 305
2012/13	2	19	21	29	87	116	195	187	382	237	191	428	1 011 657	3 083 202	4 177 965
2013/14	2	11	13	18	45	63	110	112	222	118	112	230	1 007 098	3 050 182	4 119 027
2014/15	1	21	22	29	131	160	140	235	375	146	246	392	1 568 988	4 034 667	5 708 912
2015/16	4	31	35	17	71	88	79	160	239	115	328	443	1 559 733	4 072 528	5 754 807

SARI, severe acute respiratory infection.

**Table 4:** Incidence of SARI and influenza‐associated SARI by virus (sub)type and paediatric and adult age group per 100 000 population in Romania in 2011/12‐2015/16 influenza seasons

	Influenza‐associated SARI incidence per 100.000 (95% CI) by virus (sub)type and age group						SARI incidence per 100.000 (95% CI) by age group		
	A(H1N1)pdm09	A(H3)	B	<16 y	≥16 y	Total	<16 y	≥16 y	Total
2011/12	‐	2.1 (1.6‐2.8)	0 (0‐2.5)	4.4 (2.9‐6.6)	1.3 (0.9‐2)	2.1 (1.6‐2.8)	24.9 (21.3‐29)	3.8 (3‐4.8)	8.8 (7.7‐9.9)
2012/13	2.1 (0‐2.7)	0.1 (0‐0.8)	1 (0.7‐1.5)	3.5 (2.2‐5.4)	2.9 (2.2‐3.7)	3.1 (2.5‐3.9)	23.4 (20.1‐27.3)	6.2 (5.2‐7.4)	10.2 (9.2‐11.5)
2013/14	0.8 (0‐1.2)	0.9 (0.5‐1.3)	‐	1.9 (1‐3.6)	1.5 (1‐2.2)	1.6 (1.2‐2.2)	11.7 (9.4‐14.7)	3.7 (2.9‐4.6)	5.6 (4.8‐6.5)
2014/15	0.9 (0‐1.3)	0.9 (0.6‐1.2)	1.2 (0.9‐1.6)	1.9 (1.2‐3.1)	3.4 (2.8‐4.2)	2.9 (2.4‐3.5)	9.3 (7.6‐11.4)	6.1 (5.2‐7.1)	6.9 (6.1‐7.7)
2015/16	2.6 (0‐3.2)	0 (0‐0.8)	0.2 (0.1‐0.4)	1.6 (0.9‐2.7)	3.6 (2.9‐4.4)	2.8 (2.3‐3.4)	7.4 (5.9‐9.3)	8.1 (7.1‐9.2)	7.7 (6.9‐8.6)

SARI, severe acute respiratory infection.

95% CI: 95% confidence interval.

Data on the rate of influenza in pregnant women with ILI were only available for the 2015/2016 influenza season. Of the 156 influenza‐positive ILI cases in 2015/16, 4 of 97 (5%) occurred in pregnant women (none of them vaccinated), and 31 of 156 (20%) in patients who were suffering from the underlying medical conditions. Among influenza‐positive SARI cases during 2011/2012‐2015/2016, approximately 1%‐7% were pregnant and 51%‐66% suffered from underlying medical conditions. None of the pregnant women were vaccinated, and approximately 2% of those suffering from the underlying medical conditions were vaccinated. It was not possible to calculate the incidence for these groups as the catchment population for these target groups was not known.

## 4 DISCUSSION

Our results suggest that influenza is associated with substantial morbidity in terms of primary care attendances due to ILI and hospitalizations due to SARI in Romania. In total, at least 13 621‐63 373 ILI cases and 1118‐2059 hospitalizations due to SARI were estimated to occur annually in Romania during different influenza seasons.

The highest influenza‐associated ILI incidence estimates of 70‐395 cases per 100 000 depending on the influenza season were found in children aged 0‐4 years. Except for the 2014/15 influenza season when both influenza A and influenza B were circulating, children aged 0‐4 years had the highest incidence and more influenza‐associated ILI visits than those aged 5‐14 years, which was consistent with previous estimates coming from Europe and the USA.^14^, ^15^ Due to differences in surveillance systems, case definitions, access to care and healthcare‐seeking behaviour, direct comparisons of the incidence rates are not possible; however, similar trends were observed. As in previous reports,^16^, ^17^ the incidence of SARI was higher among younger (<16 years old) than older cases (≥16 years old) except for 2015/16 season, a season dominated by A(H1N1)pdm09 when the incidence of SARI was higher in the older cases (≥16 years old). The incidence of influenza‐associated SARI was similar between the age groups for all seasons except 2011/2012, when the rate in the <16‐year‐old group was 4 times higher than the older group. Overall, there was higher incidence of influenza‐related ILI in 2014/2015 season with cocirculation of all 3 viruses with increased incidence in all age groups.

Our results showed that the CFP of hospitalized laboratory‐confirmed cases varied from 1% in the 2011/12 season to 40% in the 2015/16 with a median of 18%. This is similar to what was reported by^18^ from a study of SARI in the WHO European Region from 2009/2010 to 2011/2012 where 6 countries (Albania, Armenia, Georgia, Kazakhstan, Romania, and Ukraine) report CFP for laboratory‐confirmed SARI cases^18^. The CFP varied from 0%‐70%, but in over half of the seasons, the CFP was <2%. The high CFP of 14%‐40% seen in this study may represent all SARI cases; however, it is also possible that participating clinicians were more likely to test severe or critical cases, which inflated the estimates. The CFP for SARI cases in this study is similar to what has been reported from influenza SARI and all influenza cases in intensive care units (ICU) in other countries in Europe. In Spain, the CFP in laboratory‐confirmed SARI ICU cases for 2011/2012‐2013/2014 seasons was 15%,^19^ while in France the CFP of laboratory‐confirmed influenza cases in the ICU in 2010/2011‐2012/2013 ranged from 16%‐19%,^20^ and in Greece, during the 2010/2011 (post‐pandemic) season, the CFP was 39%.^21^ This could be explained by more patients being recruited in critical condition, when they were more likely to die, while others, less severe, might not have been captured; however, this does not explain the large variability between seasons.

Using the Romanian influenza sentinel system, we were able to estimate clinical burden of ILI and SARI, including the proportion attributable to influenza. In order to compare influenza disease burden to other countries in the region, we therefore used methodology proposed by the WHO^10^ to estimate influenza burden in Romania.

**Table 5:** Hospital CFP among influenza‐associated laboratory‐confirmed SARI cases by year in Romania in 2011/12‐2015/16 influenza seasons

Influenza season	CFP %(95% CI)	CFP % (95% CI)	CFP % (95% CI)
	<16 y	≥16 y	Total
2011/12	3.2 (0.1‐16.7)	0 (0‐10.9)	1.4 (0‐7.8)
2012/13	6.9 (0.8‐22.8)	21.8 (13.7‐32)	18.1 (11.6‐26.3)
2013/14	11.1 (1.4‐34.7)	24.4 (12.9‐39.5)	20.6 (11.5‐32.7)
2014/15	3.4 (0.1‐17.8)	16 (10.2‐23.5)	13.8 (8.8‐20.1)
2015/16	23.5 (6.8‐49.9)	43.7 (31.9‐56)	39.8 (29.5‐50.8)

CFP, case fatality proportion; SARI, severe acute respiratory infection.

95% CI: 95% confidence interval.

### 4.1 Limitations

This study sought to estimate the burden of influenza at public healthcare facilities in Romania. Persons who went to private clinics or hospitals were not assessed by the national influenza surveillance system. The true number of persons who did not seek care under the universal health insurance scheme in Romania is unknown, but estimated to be <20% of the population (personal communication, Odette Popovici, 2017). Therefore, our findings are likely to be an underrepresentation of the true medically attended ILI burden of disease in Romania by up to 20% of ILI cases.

This study relied on data from the national influenza sentinel surveillance system, which has been collecting ILI and SARI data in Romania since 2009. These data were collected for public health purposes relying on data voluntarily collected from clinicians to detect and report cases in addition to their normal duties, and may be limited in comparison with data collected in targeted research programmes. Although steps were taken to minimize potential bias, the surveillance system consisted of many doctors from numerous sites. Differences in adherence to protocols, hospitalization practices and patient healthcare‐seeking behaviour could not be accounted for in the analysis.

Samples for influenza testing for ILI cases were only collected from patients who belonged to the risk groups targeted for vaccination in Romania. In this analysis, we used the per cent of the influenza‐positive ILI cases from these vaccine target groups to estimate the influenza positivity for all ILI cases. If these cases from the vaccine target groups were more likely to be influenza positive on testing, then we may have overestimated the ILI rates. Unfortunately, it was not possible to determine the incidence in the risk groups as the Romanian population for each risk group was not known. Also, the number of pregnant women in the surveillance sites was very low, so we were not able to estimate influenza burden in this specific group. Although influenza has been shown to cause severe disease at higher incidence in the very young and the elderly,^7^ we were not able to examine the incidence in these age groups. We were only able to calculate the SARI incidence for children less than 16 years of age and all adults (≥16 years old), because hospital discharge data were only available for these groups. In addition, very low vaccination uptake rates among the cases registered by the surveillance system did not allow us to estimate influenza burden between the vaccinated and the unvaccinated individuals.

During the study period, two changes were made to the sentinel surveillance system protocol. Starting in the 2014/2015 season, the number of primary care sites participating in influenza surveillance decreased to 195 GPs. While the absolute number of ILI and influenza‐associated ILI were impacted by the change in the protocol, it is unlikely to have impacted the rates. The case definition for SARI changed in 2013/2014 expanding the time since the onset of disease to 10 days. This could have allowed more people to be considered as SARI cases, especially as there may be a delay from onset until a person is admitted to hospital. However, this change in case definition does not appear to have resulted in an overall increase SARI or influenza‐associated SARI incidence.

Only the first 2 patients presenting with ILI symptoms every Tuesday at each primary care site and 1‐3 cases with SARI per hospital were sampled. This may not have been a sufficient number to adequately represent the relative intensity of influenza activity and to refine per cent positive influenza cases needed for estimation of the influenza‐associated ILI and SARI in Romania, especially during the height of the influenza season when the relative sampling fraction is lowest.

A systematic evaluation of the national sentinel surveillance system would provide information on adherence to case definitions and protocol, optimization of sampling strategy to meet system objectives and system quality. This enhanced understanding of the sentinel surveillance system data would assist when future studies are performed to assess influenza burden in the groups targeted for influenza vaccination in Romania and the cost‐effectiveness of influenza vaccination.

## 5 CONCLUSION

This evaluation using the recently proposed methodology by WHO^10^ was the first study estimating influenza burden in Romania. We found that during each influenza season, a substantial number of persons in Romania suffer from influenza‐related ILI or are hospitalized due to influenza‐associated SARI.

This information will be used to support influenza prevention campaigns during the influenza season in Romania to improve vaccination uptake and increase awareness of influenza prevention and treatment.

## CONFLICT OF INTERESTS

GG, AP, RP, OP, DC, MJ and DG have no conflict of interest to declare. CDs appointment is partly supported by grants of different pharmaceutical companies, including companies involved in the subject matter of this study.

## APPENDIX 1

### Incidence of influenza by virus (sub)type and age group and ILI incidence by age group in Romanian population in 2011/12‐2015/16 influenza seasons

**Table:**

Season	Influenza‐associated ILI incidence in the Romanian population (95% CI)								ILI incidence in the Romanian population (95% CI)					Total population in Romania
	A(H1N1)pdm09	A(H3)	B	0‐4 y	5‐14 y	15‐64 y	≥65 y	Total	0‐4 y	5‐14 y	15‐64 y	≥65 y	Total	
2011/12	‐	6397 (5355‐7640)	38 (0‐6879)	1058 (886‐1263)	966 (809‐1153)	3458 (2897‐4128)	435 (365‐520)	6435 (5390‐7682)	4226 (3541‐5042)	3759 (3115‐4536)	12 035 (10 675‐13 570)	2183 (2168‐2198)	23 765 (21 908‐25 781)	20 199 059
2012/13	2443 (1660‐3596)	172 (19‐1567)	4672 (3521‐6200)	803 (88‐7329)	1329 (146‐12 138)	4406 (483‐40 228)	313 (34‐2856)	7287 (798‐66 536)	3154 (2557‐3890)	3475 (2840‐4251)	11 095 (9792‐12 572)	1102 (1094‐1110)	19 957 (18 248‐21 825)	20 095 996
2013/14	1510 (1010‐2258)	3048 (2302‐4037)	92 (10‐839)	677 (545‐840)	562 (453‐698)	2964 (2388‐3679)	308 (248‐383)	4684 (3774‐5813)	2257 (1749‐2912)	1786 (1329‐2401)	8043 (6948‐9312)	979 (972‐986)	13 621 (12 217‐15 186)	20 020 074
2014/15	8195 (5828‐11 525)	13959 (10996‐17 720)	15 552 (11 877‐20 365)	3824 (3012‐4854)	8489 (6687‐10 776)	20 281 (15 977‐25 746)	2466 (1943‐3131)	37 550 (29 581‐47 668)	7251 (6051‐8689)	15 560 (13 843‐17 490)	32 318 (29 518‐35 384)	4594 (4553‐4635)	63 373 (59 505‐67 491)	19 953 089
2015/16	10 570 (8475‐13 182)	1086 (558‐2115)	2359 (1152‐4830)	1998 (1647‐2423)	2698 (2224‐3272)	7791 (6423‐9451)	686 (566‐833)	14 015 (11 553‐17 000)	7270 (6063‐8716)	9401 (8080‐10 939)	18 991 (16 855‐21 399)	2149 (2130‐2169)	40 656 (37 565‐44 000)	19 870 647

ILI, influenza‐like illness.95% CI: 95% confidence interval.

## APPENDIX 2

### Incidence of influenza‐associated SARI and SARI by age group in Romania in 2011/12‐2015/16 influenza seasons

**Table:**

	Influenza‐associated SARI incidence in the Romanian population (95% CI)			SARI in the Romanian population (95% CI)		
	<16 y	≥16 y	Total	<16 y	≥16 y	Total
2011/12	130 (87‐195)	230 (151‐350)	431 (329‐565)	733 (628‐856)	655 (519‐826)	1769 (1559‐2006)
2012/13	102 (65‐160)	495 (380‐643)	625 (505‐774)	687 (589‐802)	1063 (894‐1264)	2059 (1839‐2304)
2013/14	56 (30‐106)	252 (171‐371)	317 (232‐434)	342 (273‐429)	628 (498‐791)	1118 (956‐1308)
2014/15	56 (34‐91)	579 (471‐712)	584 (485‐704)	271 (222‐331)	1039 (893‐1208)	1370 (1217‐1541)
2015/16	46 (27‐80)	606 (496‐741)	563 (466‐680)	214 (170‐269)	1367 (1200‐1556)	1530 (1369‐1709)

SARI, severe acute respiratory infection.95% CI: 95% confidence interval.
